# Purification and Characterization of a Collagenolytic Enzyme from a Pathogen of the Great Barrier Reef Sponge, *Rhopaloeides odorabile*

**DOI:** 10.1371/journal.pone.0007177

**Published:** 2009-09-24

**Authors:** Joydeep Mukherjee, Nicole Webster, Lyndon E. Llewellyn

**Affiliations:** 1 School of Environmental Studies, Jadavpur University, Kolkata, India; 2 Australian Institute of Marine Science, Townsville, Queensland, Australia; Northern Fisheries Centre, Australia

## Abstract

**Background:**

In recent years there has been a global increase in reports of disease affecting marine sponges. While disease outbreaks have the potential to seriously impact on the survival of sponge populations, the ecology of the marine environment and the health of associated invertebrates, our understanding of sponge disease is extremely limited.

**Methodology/Principal Findings:**

A collagenolytic enzyme suspected to enhance pathogenicity of bacterial strain NW4327 against the sponge *Rhopaloeides odorabile* was purified using combinations of size exclusion and anion exchange chromatography. After achieving a 77-fold increase in specific activity, continued purification decreased the yield to 21-fold with 7.2% recovery (specific activity 2575 collagen degrading units mg^−1^protein) possibly due to removal of co-factors. SDS-PAGE of the partially pure enzyme showed two proteins weighing approximately 116 and 45 kDa with the heavier band being similar to reported molecular weights of collagenases from *Clostridium* and marine *Vibrios*. The enzyme degraded tissue fibres of several sponge genera suggesting that NW4327 could be deleterious to other sponge species. Activity towards casein and bird feather keratin indicates that the partially purified collagenase is either a non-selective protease able to digest collagen or is contaminated with non-specific proteases. Enzyme activity was highest at pH 5 (the internal pH of *R. odorabile*) and 30°C (the average ambient seawater temperature). Activity under partially anaerobic conditions also supports the role of this enzyme in the degradation of the spongin tissue. Cultivation of NW4327 in the presence of collagen increased production of collagenase by 30%. Enhanced enzyme activity when NW4327 was cultivated in media formulated in sterile natural seawater indicates the presence of other factors that influence enzyme synthesis.

**Conclusions/Significance:**

Several aspects of the sponge disease etiology were revealed, particularly the strong correlation with the internal tissue chemistry and environmental temperature. This research provides a platform for further investigations into the virulence mechanisms of sponge pathogens.

## Introduction

Reports of sponge disease are rapidly increasing with Mediterranean [1] and Caribbean [2] sponge populations most heavily affected. Reports also suggest an increased prevalence of disease in sponges from Papua New Guinea [3], the Great Barrier Reef [4] and Mexico [5]. Disease outbreaks have the potential to seriously impact on the survival of sponge populations, the ecology of the marine environment and the health of associated invertebrates [6].

Knowledge of sponge disease dynamics (including the causative agents, modes of transmission and pathogen virulence mechanisms) is extremely limited. To date, only one study has confirmed Koch's postulates by verifying that an α -proteobacterium (strain NW4327) was the primary pathogen of the Great Barrier Reef sponge *Rhopaloeides odorabile* [4]. Sponges infected with strain NW4327 exhibited high levels of tissue necrosis and bacteria were observed burrowing through the collagenous spongin fibres. As further evidence of its involvement in spongin fibre necrosis, strain NW4327 degraded commercial preparations of azo-collagen. To verify the presence of a collagenolytic enzyme we have partially purified the enzyme from pathogen NW4327 and characterize its properties in relation to the disease process,

## Materials and Methods

### 2.1. Culture conditions

A stock culture of the bacterium NW4327 [4] was maintained on solid Difco Marine Agar 2216 at 0–4°C and sub-cultured every month. For large scale enzyme preparation, the isolate was grown in 2 litres of liquid medium (Difco Marine Broth 2216, pH 7.5 to 8.0) formulated in deionised and purified water (∼18 MΩ) using a MilliQ system (Millipore, Sydney, Australia) and incubated in a rotary shaker set at 28°C and 100 rpm for 48 hrs. The cell mass was separated by centrifugation (Beckman Coulter Avanti J26 XPI at 10,000 rpm for 15 min using a JLA 16.250 rotor) and the supernatant preserved for further purification by storing at 0–4°C.

### 2.2. Assay of collagenase activity

#### 2.2.1. Rapid assay using Azocoll

For rapid determination of collagenase activity the procedure described by Chavira and colleagues [7] was followed. Azocoll (Sigma Aldrich, Sydney, Australia) substrate was prepared in 50 mM Tris HCl with 1 mM CaCl_2_, pH 7.8. Unless otherwise stated, 500 µl of the suspension was incubated with 500 µl of test sample at 37°C for 2 hours with shaking in a Jitterbug™ microtitre plate thermal shaker (Model 130000, Boekel Scientific, PA, USA). The reaction was stopped by immersing the samples in ice for three minutes and the unreacted azocollagen was removed by centrifugation (Eppendorf AG Model 5810R centrifuge at 3,000 rpm for 10 min in a A-2 DWP rotor). Absorbance of the supernatant (200 µl) containing the azo-labelled peptidic digestion products was measured at 520 nm in a Synergy HT plate reader (BioTek, Vermont, USA) with increased absorbance values indicating higher collagenase activity.

#### 2.2.2. Sensitive assay using ninhydrin

For sensitive determination of ultra-low collagenase activity in the chromatography fractions and calculation of collagen digestion units (CDU) the following procedure was employed: collagenase or test sample (500 µl) was incubated for 5 hours with bovine Achilles tendon collagen (500 µl) (Sigma-Aldrich) dissolved in 50 mM Tris HCl with 1 mM CaCl_2_, pH 7.8. An aliquot (300 µl) of the reaction mixture was withdrawn, 60 µl of 0.05 M EDTA added and chilled in ice to stop the reaction. 400 µl of ninhydrin reagent [8] was added and the mixture heated at 100°C for five min. Finally, 1.0 ml of a diluent was added and absorbance read at 570 nm. One CDU is defined as the amount of enzyme that liberates peptide, equivalent to 1 mol leucine,from collagen in 5 hours at pH 7.5 and 37°C.

### 2.3. Microbial growth measurement

Microbial growth was measured by quantifying either extracellular protein in the media or intracellular protein released after sonication. Intracellular protein was measured after pelleting a 1.0 ml aliquot of cultures using centrifugation, resuspending cells in deionised and purified water (1.0 mL) and then sonication at 50 kHz in 5 sec pulses for 5 min using a Cole Parmer Model 130W ultrasonic processor. Protein concentrations were measured using the Bio-Rad Protein Assay according to the manufacturer's protocol (Bio Rad Laboratories, USA) which is based on the method of Bradford [9].

### 2.4. Purification of collagenase

#### 2.4.1. Ultrafiltration

The crude culture filtrate was ultrafiltered through a 50 kDa ultrafiltration membrane at room temperature (∼22–24°C) (Pall Corporation, USA) and the retentate used for chromatography. Ultrafiltration was conducted in an Amicon Model 52 (Cell capacity 50.0 mL; membrane diameter 43 mm) stirred ultrafiltration cell with pressure applied using high purity nitrogen gas (BOC Australia, Townsville, Australia).

#### 2.4.2. Semi-preparative molecular size exclusion chromatography

The ultrafiltration retentate was applied to a 1×100 cm column packed with Superdex G200 (GE Healthcare, Sydney, Australia) which was eluted with 10 mM Tris HCl and 4 mM CaCl_2_, pH 9.0, [10] flowing at 0.3 ml min^−1^ with fractions collected every 13 min.

#### 2.4.3. Cation exchange chromatography

Active fractions from the semi-preparative size-exclusion chromatography as determined using the rapid Azocoll assay were pooled and concentrated by ultrafiltration through a 10 kDa membrane (Pall Corporation, USA). Portions (500 µl) of the concentrate were then applied to a Resource Q (GE Healthcare, Sydney, Australia) column (1×6 cm) attached to a Shimadzu Class VP HPLC system. Gradient elution was carried out between buffer A (10 mM Tris-HCl, 1 mM CaCl_2_, pH 8.5) and buffer B (10 mM Tris HCl, 1 mM CaCl_2_, pH 8.5 with 0.5 M NaCl), at a flow rate of 1.0 ml min^−1^ with fractions collected every minute. The gradient profile was: 0–10 mins-100% A; 10–25 mins-100%A to 100%B; 25–30 mins-100%B; 30–35 mins-100%B to 100%A). The column eluate was monitored with a photodiode array (PDA) detector set at 280/254 nm. Collagenase activity was measured using the previously described and more sensitive ninhydrin method with active fractions pooled and concentrated by ultrafiltration through a 50 kDa membrane (Pall Corporation, USA) prior to analytical molecular size exclusion chromatography.

#### 2.4.4. Analytical molecular size exclusion chromatography (Superdex 200HR)

Active fractions from the Resource Q column were applied to a GE Healthcare pre-packed gel filtration column (Superdex 200 HR 10/30 column) attached to the Shimadzu Class VP HPLC system with PDA detector set at 280/254 nm. The column was eluted with 10 mM Tris HCl with 4 mM CaCl_2_, pH 9.0, at a flow rate 1.0 ml min^−1^ [10].

### 2.5. Sodium dodecyl sulfate polyacrylamide gel electrophoresis (SDS-PAGE)

SDS-PAGE was performed in BioRad Criterion (Bio Rad Laboratories, USA) precast gels at a constant 200 V for 55 minutes. Initial staining was performed using LavaPurple [11]. The manufacturer's (Fluorotechnics, Sydney, Australia) recommended protocol (Total Staining for Gels and Blots) was followed and the bands were visualized in a Vilber Lourmat Chemi-Smart 3000 imager.

### 2.6. Substrate specificity

Specificity of the collagenase enzyme from NW4327 for *Rhopaloeides odorabile* spongin fibres was assessed by measuring its ability to degrade tissue fibres from other marine sponges. Material from *Ianthella flabelliformis*, *Cacospongia* sp., *Ircinia* sp. and *Luffariella* sp. was prepared by removing the non-fiber components by thorough rinsing with running tap water and homogenizing the remaining air-dried tissue in a Waring blender. Specificity was further assessed using commercial casein and gelatin (Sigma-Aldrich) and bird feather keratin obtained by homogenizing the vanes cut off from the central shaft of the moulted tail feather of the bush turkey (*Alectura lathami*). Since large amount of enzyme was required for these experiments and because of an observed loss of specific activity as the protein became more pure (see Results), these substrates were digested with collagenase obtained after semi-preparative molecular size exclusion chromatography. Assays were conducted by suspending 5 mg of the above-mentioned substrates in 5 ml of 50 mM Tris HCl (pH 7.5) buffer with 1 mM CaCl_2_, as used for the sensitive ninhydrin assay, see Section 2.2.2.

### 2.7. Effect of assay pH and temperature on collagenase activity

Collagenase activity was measured using the sensitive ninhydrin protocol in the pH range 4.0 to 9.0. A three component buffer system (100 mM 2-(*N*-morpholino)ethanesulfonic acid **(**MES)/50 mM Tris/50 mM acetic acid adjusted with tetramethylammonium hydroxide or HCl to the desired pH) was used to maintain a constant ionic strength and a constancy of the chemical nature of the buffers throughout this pH range [12]. Collagenolysis at pH 7.5 was measured in the range 20 to 60°C at intervals of 10°C with samples maintained at these temperatures in a thermostatically controlled water bath.

### 2.8. NW4327 growth and collagenase production in different media formulations

NW4327 was incubated in Marine Broth 2216 (Difco) prepared in autoclaved natural seawater collected from Davies Reef (18° 5'S, 147° 4'E). The pH of the media was unadjusted and the composition was actually 2x seawater (artificial and natural). Cultivation was also attempted in Marine Broth where the pH was adjusted to 5.0, 7.0 or 9.0 prior to autoclaving. In order to test the effect of sponge tissue on growth and collagenase production, fibrous *R. odorabile* tissue (cleaned of adherent non-sponge material) was added to 50 ml of the pH adjusted Marine Broth solutions and sterilised in 250 ml Erlenmeyer flasks. The flasks were incubated in a rotary shaker at 28°C and 100 rpm for 48 h. Control flasks at the three pH values without added sponge tissue were included in the experiment.

### 2.9. Effect of partial anaerobic conditions on growth and collagenase production of NW4327

Partially anaerobic conditions were created by flushing the 250 ml Erlenmeyer flask containing 50.0 ml Marine Broth 2216 prepared in MilliQ water (pH 7.0) with nitrogen gas (BOC Australia, Townsville, Australia) and adding FeS [13] as a reducing agent. The flasks were incubated in a rotary shaker at 28°C and 100 rpm for 48 h. Control flasks under normal aerobic conditions with FeS were used. All determinations in sections 2.6 to 2.9 of growth and collagenase production were performed twice in duplicate sets and the average of the values reported.

## Results

**Figure 1:**
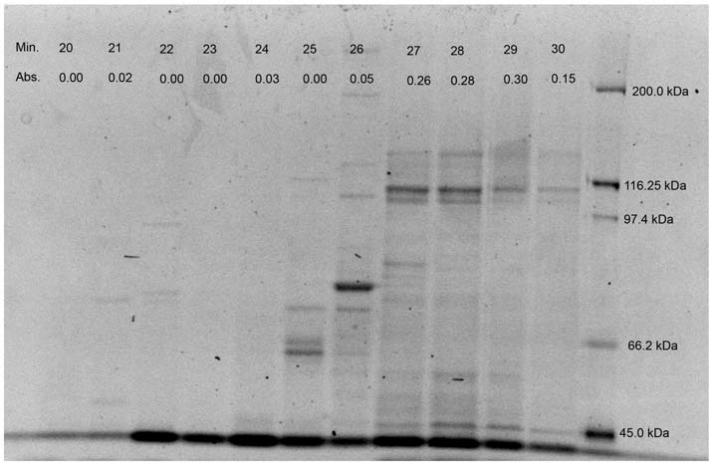
SDS PAGE analysis of fractions obtained at 20 to 30 minutes during anion exchange chromatography. Lane numbers (upper row of top panel) indicate the time in minutes (Min.) and lower row in top panel indicate Abs_570_ values obtained after assaying 500 µL of the fractions by the sensitive ninhydrin assay (Abs.). Values on the right panel indicate molecular weights of the standard protein markers.

**Figure 2:**
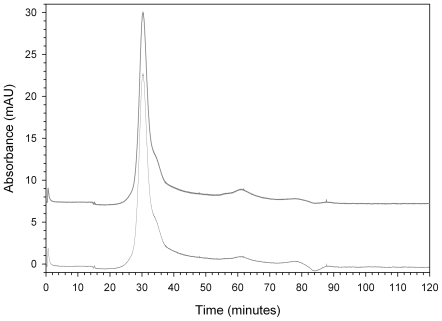
HPLC chromatogram obtained with analytical molecular size exclusion chromatography using Superdex 200 HR. The chromatograms depicted show the absorbance of the eluted material at 254 nm (lower trace) and at 280 nm (upper trace). Note that the absorbance values at 280 nm have been offset by 7.5 mAU to distinguish it from the values at 254 nm.

The first two steps of purification increased purity 77-fold (Specific activity 9414.6 CDU mg^−1^ protein) but specific activity decreased in subsequent steps down to 21-fold with 7.2% recovery, the specific activity being 2574.7 CDU mg^−1^ protein. This was despite an apparent decrease in complexity after cation exchange chromatography according to SDS-PAGE (Figure 1). The low enzyme recovery may have occurred due to the third and fourth steps being carried out at room temperature or loss of cofactors. The activity 401.7 CDU ml^−1^ obtained in the final step of purification was the activity found in the peak of the chromatogram (Figure 2).

The active fractions (27–30), shown in Figure 1 were dominated by two bands near the molecular weight marker 116.25 kDa and a third band near the 45 kDa range. The chromatogram obtained during the final step of purification using analytical size exclusion shows only one peak (Figure 2). SDS-PAGE analysis of this final sample, however, yielded two bands near the 116.25 kDa region similar to the bands observed in fraction 30 of Figure 1 (data not shown). Given the small amount of the product obtained (about 200 µl) after the four steps of the isolation process, no further methods were deemed practical to attempt additional purification of the enzyme.

Assay pH affected the activity of the NW4327 collagenase with highest activity observed at pH 5.0 compared to pH 4 or pH 6 and above. Assay temperature also affected the activity of NW4327 collagenase with higher activity observed at 30°C compared to that at 20°C or at 40°C or warmer. Partially anaerobic conditions reduced growth of NW4327 by 25% and lowered the yield of collagenase from 5227±92.7 CDU ml^−1^ to 1935±70.6 CDU ml^−1^. Replacing MilliQ water with natural seawater when formulating the growth media enhanced microbial growth by 20% and collagenase production by 30% at the end of the five day cultivation. Growth of the isolate NW4327 was observed at pH 5.0 and pH 7.0 (in Marine Broth prepared in natural seawater, ie 2× seawater concentration) but not at pH 9.0. A neutral pH favoured growth while adding sponge collagen almost doubled collagenase production. The enzyme was equally able to degrade all of the sponge fibres, gelatin, casein and bird feather under the described conditions.

## Discussion

Collagen is the major fibrous component of vertebrate extracellular connective tissue such as skin, tendon, blood vessels and bone and its importance to invertebrates is increasingly apparent [14], [15], [16]. Collagen molecules are structural extracellular macromolecules containing at least one domain with a characteristic triple helical conformation. This study aimed to characterize and purify an extracellular collagenase which attacks the collagen fibres of marine sponges. Fourier Transform Infrared Reflection-Absorption [17] suggests that properties of novel invertebrate collagenases can be observed using mammalian collagen as a substrate.

True collagenase may cleave simultaneously across all three chains or attack a single strand of the collagen macromolecule. This contrasts with other collagenases which split collagen in its native triple-helical conformation at a specific site yielding fragments. Bacterial collagenases, often from pathogenic strains, differ from mammalian collagenases in that they attack many sites along the helix. Although the most studied is that from *Clostridium histolyticum*, [18], [19], other bacterial sources of collagenase include *Streptomyces lavendulae* [20], *Bacillus subtilis* [21], *Vibrio alginolyticus* [22], *Streptococcus gordonii* [23] and *Thermoactinomycetes sp*. [10]. Less studied are marine microorganisms, especially pathogens. Previous studies on collagenases from marine bacteria have adopted a general screening approach [24]. Marine bacterial collagenases isolated from other sources include *Vibrio* B-30 ATCC 21250 [25], *Vibrio vulnificus* CYK279H [26] and *E. coli* JM83 [27]. Our study, reports the purification of the collagenolytic enzyme from a marine bacterium isolated from a diseased sponge.

Commercially available *Clostridium histolyticum* collagenase preparations possess varied degrees of purity [19]. Although *C. histolyticum* Strain JCM 1403 produced only a 116-kDa polypeptide with collagenase activity under the condition used, a 98-kDa gelatinase and other polypeptides with potent gelatinolytic activity remained in the preparation. The authors concluded that it was not possible to obtain *C. histolyticum* collagenase free of gelatinase and other nonspecific proteases either from commercial sources or from *C. histolyticum* cultures. Similar observations were made by Merkel and Dreisbach [25]. The collagenase from NW4327 was also difficult to purify due to the close bands in SDS-PAGE at 116.25 kDa.

Activity towards casein and bird feather keratin indicates that the partially purified collagenase may be a highly active protease. Enzyme activity was greater at pH 5.0, the internal tissue pH of *R. odorabile* [28], compared to pH 4 or pH 6 and above. Enzymolysis was greater at 30°C than at 20°C or ≥40°C. The average sea water temperature along the Great Barrier Reef is also close to 30°C for much of the year [29]. These observations suggest a role for this enzyme in the degradation of the spongin tissue of the infected sponges. The observed pH and temperature optima are distinct from the known values obtained from another marine bacterium, *Vibrio vulnificus* CYK279H [26] and *E. coli* JM83 [27]. Although the isolated collagenase is able to degrade sponge fibres from many species, it may have the potential to express a pathogenic effect in *Rhopaloeides odorabile* due to the sponge's tissue conditions being optimum for the collagenase's activity. Similar to the observation of Merkel et al. [25], the presence of collagen enhances the activity of NW4327 collagenase.

Enhanced enzyme activity when the microorganism was cultivated in natural seawater suggests the presence of other factors not available in the Marine Difco 2216 medium influencing enzyme synthesis although an effect due to increased concentration of sea water salts in the natural seawater based media is also possible.

In conclusion, during partial purification of the collagenase enzyme from the sponge pathogen NW4327, several aspects of the sponge disease etiology were elucidated; namely the strong correlation with the internal tissue chemistry and environmental temperature. This research provides a platform for further investigations into the virulence mechanisms of sponge pathogens.
